# Supplementary material for: Multi‐dataset identification of innovative feature genes and molecular mechanisms in keratoconus
Source: J Cell Mol Med. 2024 Sep 19;28(18):e70079. doi: 10.1111/jcmm.70079 (PMC11412914; doi:10.1111/jcmm.70079)
Supplement: Supplementary file 2 — Material S2. [file JCMM-28-e70079-s002.zip › 7.GSEA analysis/4.MYRF/KEGG_AXON_GUIDANCE.html]

Details for gene set KEGG\_AXON\_GUIDANCE[GSEA]

|  || Dataset | MYRF.group.cls#h\_versus\_l |
| Phenotype | group.cls#h\_versus\_l |
| Upregulated in class | l |
| GeneSet | KEGG\_AXON\_GUIDANCE |
| Enrichment Score (ES) | -0.37661046 |
| Normalized Enrichment Score (NES) | -1.4495065 |
| Nominal p-value | 0.026104419 |
| FDR q-value | 0.21204305 |
| FWER p-Value | 0.826 |
Table: GSEA Results Summary

  

Fig 1: Enrichment plot: KEGG\_AXON\_GUIDANCE      
 Profile of the Running ES Score & Positions of GeneSet Members on the Rank Ordered List

  

| SYMBOL | TITLE | RANK IN GENE LIST | RANK METRIC SCORE | RUNNING ES | CORE ENRICHMENT || 1 | NFATC2 | na | 1281 | 0.423 | -0.0174 | No |
| 2 | UNC5C | na | 1664 | 0.386 | -0.0105 | No |
| 3 | SEMA3E | na | 1720 | 0.381 | 0.0056 | No |
| 4 | PPP3R2 | na | 2013 | 0.356 | 0.0137 | No |
| 5 | RAC3 | na | 2468 | 0.325 | 0.0156 | No |
| 6 | UNC5A | na | 2664 | 0.313 | 0.0246 | No |
| 7 | ABLIM3 | na | 2865 | 0.302 | 0.0328 | No |
| 8 | CHP2 | na | 3253 | 0.283 | 0.0348 | No |
| 9 | EPHA5 | na | 4233 | 0.246 | 0.0179 | No |
| 10 | NTN3 | na | 4622 | 0.234 | 0.0176 | No |
| 11 | SEMA6C | na | 4907 | 0.225 | 0.0198 | No |
| 12 | EPHA6 | na | 4926 | 0.225 | 0.0297 | No |
| 13 | EPHA4 | na | 5047 | 0.221 | 0.0365 | No |
| 14 | EPHB1 | na | 5199 | 0.216 | 0.0422 | No |
| 15 | SEMA5B | na | 6671 | 0.173 | 0.0077 | No |
| 16 | RGS3 | na | 7506 | 0.151 | -0.0094 | No |
| 17 | ROBO2 | na | 7780 | 0.144 | -0.0106 | No |
| 18 | EPHA8 | na | 8093 | 0.136 | -0.0133 | No |
| 19 | ABLIM2 | na | 8644 | 0.124 | -0.0234 | No |
| 20 | ROCK1 | na | 8751 | 0.121 | -0.0209 | No |
| 21 | ABLIM1 | na | 8756 | 0.121 | -0.0154 | No |
| 22 | NCK2 | na | 8807 | 0.120 | -0.0112 | No |
| 23 | SRGAP3 | na | 8849 | 0.119 | -0.0069 | No |
| 24 | EFNA2 | na | 8967 | 0.117 | -0.0048 | No |
| 25 | KRAS | na | 9371 | 0.108 | -0.0115 | No |
| 26 | RHOD | na | 9580 | 0.104 | -0.0126 | No |
| 27 | RASA1 | na | 9714 | 0.101 | -0.0118 | No |
| 28 | GNAI3 | na | 9952 | 0.097 | -0.0141 | No |
| 29 | NTN1 | na | 9995 | 0.096 | -0.0109 | No |
| 30 | SEMA4C | na | 10486 | 0.087 | -0.0210 | No |
| 31 | LIMK1 | na | 10531 | 0.086 | -0.0183 | No |
| 32 | PLXNA1 | na | 10549 | 0.086 | -0.0148 | No |
| 33 | EPHA1 | na | 11059 | 0.077 | -0.0259 | No |
| 34 | SEMA4D | na | 11206 | 0.075 | -0.0267 | No |
| 35 | SEMA3F | na | 11232 | 0.075 | -0.0239 | No |
| 36 | PLXNA2 | na | 11996 | 0.064 | -0.0430 | No |
| 37 | ROBO1 | na | 13434 | 0.045 | -0.0825 | No |
| 38 | ROCK2 | na | 13790 | 0.040 | -0.0909 | No |
| 39 | MAPK3 | na | 14382 | 0.034 | -0.1064 | No |
| 40 | SEMA6D | na | 14419 | 0.033 | -0.1059 | No |
| 41 | UNC5B | na | 14467 | 0.033 | -0.1057 | No |
| 42 | LRRC4C | na | 14752 | 0.030 | -0.1125 | No |
| 43 | GNAI2 | na | 15256 | 0.025 | -0.1259 | No |
| 44 | EPHB3 | na | 16157 | 0.016 | -0.1511 | No |
| 45 | EFNA5 | na | 16428 | 0.013 | -0.1583 | No |
| 46 | EPHA3 | na | 17153 | 0.007 | -0.1789 | No |
| 47 | RAC2 | na | 17259 | 0.006 | -0.1816 | No |
| 48 | SEMA4A | na | 17724 | 0.002 | -0.1949 | No |
| 49 | PPP3R1 | na | 17799 | 0.001 | -0.1970 | No |
| 50 | PAK4 | na | 17893 | 0.001 | -0.1997 | No |
| 51 | SEMA3B | na | 17983 | -0.000 | -0.2023 | No |
| 52 | PLXNB2 | na | 18184 | -0.002 | -0.2079 | No |
| 53 | RHOA | na | 18827 | -0.008 | -0.2261 | No |
| 54 | CFL1 | na | 19157 | -0.012 | -0.2350 | No |
| 55 | CHP1 | na | 19165 | -0.012 | -0.2347 | No |
| 56 | PAK3 | na | 19225 | -0.013 | -0.2358 | No |
| 57 | GSK3B | na | 19466 | -0.016 | -0.2420 | No |
| 58 | EFNA4 | na | 19897 | -0.020 | -0.2534 | No |
| 59 | SEMA3G | na | 20048 | -0.022 | -0.2567 | No |
| 60 | CDK5 | na | 20160 | -0.024 | -0.2588 | No |
| 61 | EPHB4 | na | 20281 | -0.025 | -0.2611 | No |
| 62 | SRGAP1 | na | 20393 | -0.026 | -0.2631 | No |
| 63 | MAPK1 | na | 20406 | -0.027 | -0.2622 | No |
| 64 | PLXNB1 | na | 20962 | -0.033 | -0.2767 | No |
| 65 | NFATC3 | na | 21139 | -0.035 | -0.2802 | No |
| 66 | SLIT1 | na | 21204 | -0.036 | -0.2804 | No |
| 67 | EFNA1 | na | 21546 | -0.040 | -0.2884 | No |
| 68 | EPHA7 | na | 21685 | -0.042 | -0.2904 | No |
| 69 | ARHGEF12 | na | 21776 | -0.043 | -0.2910 | No |
| 70 | PAK6 | na | 21811 | -0.043 | -0.2900 | No |
| 71 | SEMA6B | na | 21831 | -0.044 | -0.2885 | No |
| 72 | HRAS | na | 21856 | -0.044 | -0.2872 | No |
| 73 | RAC1 | na | 21936 | -0.045 | -0.2873 | No |
| 74 | PPP3CA | na | 22219 | -0.049 | -0.2932 | No |
| 75 | SEMA4B | na | 22290 | -0.049 | -0.2930 | No |
| 76 | DPYSL5 | na | 22562 | -0.053 | -0.2983 | No |
| 77 | PPP3CB | na | 22763 | -0.056 | -0.3015 | No |
| 78 | ROBO3 | na | 22926 | -0.058 | -0.3035 | No |
| 79 | CXCL12 | na | 22943 | -0.059 | -0.3012 | No |
| 80 | CDC42 | na | 23297 | -0.064 | -0.3085 | No |
| 81 | PLXNC1 | na | 23331 | -0.065 | -0.3064 | No |
| 82 | PTK2 | na | 23650 | -0.070 | -0.3124 | No |
| 83 | DCC | na | 23704 | -0.071 | -0.3106 | No |
| 84 | EFNB1 | na | 23708 | -0.071 | -0.3074 | No |
| 85 | ABL1 | na | 23912 | -0.074 | -0.3098 | No |
| 86 | PAK2 | na | 24412 | -0.082 | -0.3204 | No |
| 87 | LIMK2 | na | 24514 | -0.084 | -0.3194 | No |
| 88 | EPHB2 | na | 24915 | -0.092 | -0.3267 | No |
| 89 | NCK1 | na | 25117 | -0.095 | -0.3281 | No |
| 90 | PLXNB3 | na | 25501 | -0.103 | -0.3344 | No |
| 91 | SRGAP2 | na | 25541 | -0.103 | -0.3307 | No |
| 92 | SEMA4G | na | 26185 | -0.117 | -0.3439 | No |
| 93 | PAK1 | na | 26325 | -0.120 | -0.3423 | No |
| 94 | EFNA3 | na | 26828 | -0.131 | -0.3508 | No |
| 95 | L1CAM | na | 26845 | -0.131 | -0.3451 | No |
| 96 | NFAT5 | na | 27258 | -0.141 | -0.3505 | No |
| 97 | SEMA5A | na | 27412 | -0.146 | -0.3482 | No |
| 98 | PPP3CC | na | 28351 | -0.171 | -0.3673 | Yes |
| 99 | GNAI1 | na | 28414 | -0.173 | -0.3611 | Yes |
| 100 | SEMA3A | na | 28677 | -0.181 | -0.3603 | Yes |
| 101 | MET | na | 28755 | -0.183 | -0.3540 | Yes |
| 102 | EPHB6 | na | 29127 | -0.195 | -0.3556 | Yes |
| 103 | SLIT3 | na | 29854 | -0.219 | -0.3665 | Yes |
| 104 | ITGB1 | na | 29873 | -0.219 | -0.3568 | Yes |
| 105 | PLXNA3 | na | 29884 | -0.220 | -0.3469 | Yes |
| 106 | NRAS | na | 30019 | -0.224 | -0.3403 | Yes |
| 107 | EFNB2 | na | 30375 | -0.237 | -0.3396 | Yes |
| 108 | NFATC1 | na | 31545 | -0.293 | -0.3598 | Yes |
| 109 | EPHA2 | na | 31828 | -0.308 | -0.3536 | Yes |
| 110 | UNC5D | na | 32086 | -0.322 | -0.3461 | Yes |
| 111 | EFNB3 | na | 32146 | -0.326 | -0.3326 | Yes |
| 112 | NGEF | na | 32162 | -0.327 | -0.3178 | Yes |
| 113 | NFATC4 | na | 32887 | -0.379 | -0.3212 | Yes |
| 114 | FES | na | 32907 | -0.381 | -0.3040 | Yes |
| 115 | NRP1 | na | 32909 | -0.381 | -0.2864 | Yes |
| 116 | CFL2 | na | 33257 | -0.414 | -0.2772 | Yes |
| 117 | SLIT2 | na | 33371 | -0.428 | -0.2606 | Yes |
| 118 | SEMA3D | na | 33435 | -0.435 | -0.2422 | Yes |
| 119 | NTNG1 | na | 33813 | -0.489 | -0.2304 | Yes |
| 120 | NTN4 | na | 33837 | -0.493 | -0.2082 | Yes |
| 121 | DPYSL2 | na | 33868 | -0.499 | -0.1859 | Yes |
| 122 | FYN | na | 33971 | -0.517 | -0.1648 | Yes |
| 123 | SEMA6A | na | 34029 | -0.528 | -0.1420 | Yes |
| 124 | SEMA4F | na | 34063 | -0.534 | -0.1181 | Yes |
| 125 | RND1 | na | 34179 | -0.566 | -0.0951 | Yes |
| 126 | SEMA7A | na | 34473 | -0.668 | -0.0726 | Yes |
| 127 | CXCR4 | na | 34584 | -0.727 | -0.0420 | Yes |
| 128 | SEMA3C | na | 34712 | -0.993 | 0.0005 | Yes |
Table: GSEA details [plain text format]

  

Fig 2: KEGG\_AXON\_GUIDANCE      
 Blue-Pink O' Gram in the Space of the Analyzed GeneSet

  

Fig 3: KEGG\_AXON\_GUIDANCE: Random ES distribution      
 Gene set null distribution of ES for **KEGG\_AXON\_GUIDANCE**
